# Low-cost pulmonary rehabilitation for chronic respiratory diseases: a narrative review

**DOI:** 10.3389/fresc.2026.1706188

**Published:** 2026-09-11

**Authors:** Else Saliés Fonseca, Marcelo Velloso, Marcelo Couto Jorge Rodrigues, Karla Medeiros Costa, Claudio Andre Barbosa de Lira, Ana Luiza Lima Sousa, Natasha Yumi Matsunaga Spicacci, Gustavo De Conti Teixeira Costa

**Affiliations:** 1 Center for Advanced Study and Research in Sports, Federal University of Goiás, Goiânia, Brazil; 2 Laboratory for Assessment and Research in Cardiorespiratory Performance, Federal University of Minas Gerais, Belo Horizonte, Brazil; 3 Laboratory of Human and Exercise Physiology, Federal University of Goiás, Goiânia, Brazil; 4 Arterial Hypertension Unit, Federal University of Goiás, Goiânia, Brazil; 5 Functional Health Department, Federal University of Goiás, Goiânia, Brazil

**Keywords:** chronic obstructive/rehabilitation, delivery of health care, exercise therapy, health equity, physical therapist, pulmonary disease, primary healthcare, respiratory diseases

## Abstract

Pulmonary rehabilitation (PR) is a cornerstone in the management of chronic respiratory diseases (CRD), but access remains severely limited in low- and middle-income countries (LMICs) because of cost, infrastructure, and workforce barriers. This mini narrative review synthesized evidence on the effectiveness, feasibility, and implementation of low-cost PR strategies designed with minimal equipment and simplified delivery models. Low-cost PR was considered not only as exercise training using minimal or improvised equipment, but as a context-adapted rehabilitation model that preserves essential PR components while adapting to local health-system, workforce, cultural, and socioeconomic constraints. Searches were conducted in PubMed, Scopus, Web of Science, and SciELO (2014–2024); twenty publications were included, comprising randomized controlled trials, quasi-experimental studies, systematic reviews, clinical guidelines, and implementation research. Across studies, low-cost PR consistently improved exercise capacity, symptoms, and health-related quality of life, measured by 6MWT, ISWT, SGRQ, and CAT. Home-based and community programs demonstrated feasibility and safety, often comparable to conventional PR; supervision and structured education enhanced adherence, while properly instructed unsupervised programs also showed non-inferior outcomes. Tai Chi and Nordic walking emerged as culturally adaptable, low-cost alternatives. However, evidence remains heterogeneous and predominantly COPD-focused, with limited data for other CRD and few studies in primary healthcare, restricting generalizability to the broader CRD burden in LMICs. Effective scale-up requires attention to workforce availability, primary healthcare integration, contextual barriers to home-based rehabilitation, digital accessibility, education, equity, and long-term sustainability. Further high-quality trials and implementation studies are needed to consolidate evidence and inform policies that integrate context-adapted, affordable PR into healthcare systems.

## Introduction

Chronic respiratory diseases (CRD), such as chronic obstructive pulmonary disease (COPD), asthma, and interstitial lung disease, represent a growing global health burden, leading to high morbidity, mortality, and healthcare costs (1–3). Due to their high prevalence, COPD, asthma, lung cancer, and tuberculosis represent important respiratory conditions in the public health scenario (2). Individuals with CRD may experience dyspnea, reduced functional capacity, and impaired quality of life (4).

The main risk factors for CRD include smoking, exposure to pollutants and allergens, occupational risks, poor diet, obesity, and physical inactivity (2, 4). Globally, millions of individuals are affected, with a substantial burden occurring in developing countries (5). In Brazil, COPD accounted for 17% of hospitalizations among adults older than 40 years in 2018, while asthma accounted for 47,897 hospitalizations in 2020 (6). National data reported 148,411 CRD deaths in 2021 and 173,512 hospitalizations between January 2022 and January 2023 (7). In 2019, CRDs affected 454.56 million people worldwide, resulting in approximately 3.97 million deaths and 103.53 million disability-adjusted life years (1, 8–10). In Brazil, the burden associated with CRD continues to be influenced by population growth and aging (5, 10, 11).

In this context, pulmonary rehabilitation is one of the most effective non-pharmacological interventions for chronic respiratory diseases, improving symptoms such as dyspnea and fatigue, enhancing functionality, and reducing healthcare utilization (4, 12, 13). Traditionally, PR programs require specialized assessment facilities, multidisciplinary teams, and individualized plans; however, access may be restricted by costs, travel requirements, limited infrastructure, and service availability (14–16). Alternative models, including telerehabilitation, home-based rehabilitation, community-based programs, and minimal-equipment approaches, have therefore been proposed to expand access (13, 16–22).

Low-cost PR should not be interpreted solely as exercise training performed with inexpensive or improvised equipment. Rather, it may be considered a context-adapted rehabilitation approach that seeks to preserve essential PR components while adapting delivery to locally available infrastructure, workforce capacity, patient circumstances, and healthcare organization (18, 23–25). Depending on the setting, this approach may combine walking, elastic resistance, bodyweight exercise, home- or community-based delivery, hybrid monitoring, structured education, family or peer support, and task-sharing where professionally and legally appropriate (18, 23–26). This broader perspective is particularly relevant in LMICs, where barriers to PR may include limited workforce availability, fragmented referral pathways, transportation costs, geographical inequities, socioeconomic constraints, limited digital access, and discontinuity of care (16, 24, 25, 27).

Therefore, this narrative review aims to synthesize evidence on the effectiveness, feasibility, and implementation models of low-cost PR while highlighting current evidence gaps and priorities for future research.

### Search strategy

**Figure 1:**
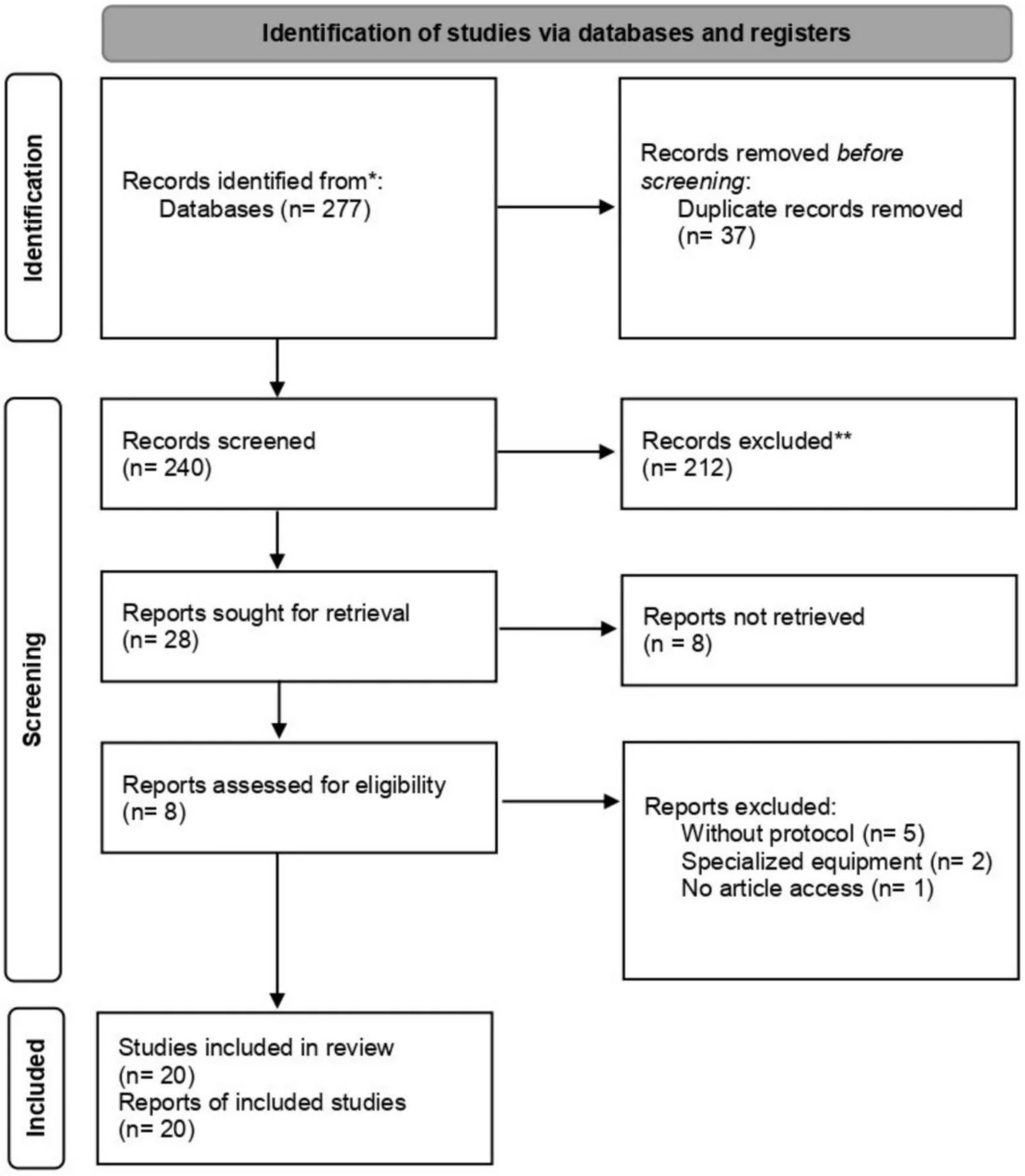
PRISMA 2020 flow diagram_Selection and review process. Source: Page MJ, et al. BMJ 2021 372:n71. doi: 10.1136/bmj.n71.

Literature searches were conducted in PubMed, Scopus, Web of Science, and SciELO for publications indexed between July 2014 and July 2024, using terms related to low-cost, minimal-equipment, or resource-adapted pulmonary rehabilitation, chronic respiratory disease, and implementation in home-based, community, or primary-care settings (search strings in Table 1). Eligible studies were peer-reviewed randomized or quasi-experimental trials, systematic reviews, implementation reports, or clinical guidelines addressing low-cost PR delivered in home-based, community, or primary-care settings, published in English or Spanish between July 2014 and July 2024; studies restricted to inpatient or highly specialized hospital-based delivery without a low-cost, community, or home-based component were excluded. Records were imported into Rayyan (Qatar Computing Research Institute, QCRI, Qatar), and screened independently by three reviewers (MCJR, KMC, ESF), with disagreements resolved by consensus (80% inter-reviewer agreement); a flow diagram summarizing identification, screening, and inclusion is provided for transparency (Figure 1). As a mini narrative review, this synthesis reflects the authors’ interpretation of the available evidence rather than a systematic or quantitative appraisal; no formal risk-of-bias assessment or meta-analysis was performed given the heterogeneity of designs, settings, and outcomes across the 20 included publications (28, 29).

**Table 1:** – Studies Characteristics

**Author, (Date) Country**	**Sample size**	**Population**	**CRD type**	**Intervention type**	**Duration**	**Outcomes**	**Key findings**
Alison JA, McKeough ZJ, 2014 (12) Australia	Review of 11 studies - 8 of endurance exercise - 3 of strength training	Stable adults	COPD	Minimal/low-cost PR (primarily walking, sit-to-stand exercise, stepping, elastic resistance band training, and Tai Chi)	4 –12 weeks 2 – 6 sessions/ week	40 m - 6MWD (95% CI: 13–67), SGRQ–7-point reduction (95% CI: –12 to –3)	Minimal-equipment PR improves capacity/HRQoL; comparable to conventional PR
Anastasaki M et al., 2019, (23) Greece	40 participants (31 completed) (77.5%) 24 – COPD, 16 - chronic asthma	Rural patients with stable CRD	COPD/asthma	Community PR with low-cost 30 minutes of outdoor walking, 1 hour of strength and resistance exercise (bands, weights, sit-to-stand, steps, static bicycle), and educational sessions	6 week (2×/week; 2 h)	Improvement for −0.53 in CCQ (95% CI −0.81 to −0.24), −5.93 in CAT (95% CI −8.27 to −3.60), −23.00 in SGRQ total (95% CI −29.42 to −16.58), reductions in PHQ-9 (−1.10; 95% CI −2.32 to 0.12)	Significant improvements in symptom and functional changes, and acceptability among patients and stakeholders, with feasibility/acceptability in primary care
Cerini et al., 2021, (29) Switzerland	53 participants	Post-PR COPD patients	COPD	HOMEX home-based resistance-training program (chair and elastic bands), coach supervision	6 days/week 20 minutes/day 12 months	Adherence and acceptability (OR 11.46; 95% CI 1.88–91.48),	70% completed; 79% ≥10 months; high acceptability.
Cheng et al., 2023, (38) Australia	Review of 19 RCTs	Adults stable/exacerbation	COPD	Minimal-equipment PR (walking, Nordic walking, stair climbing, elastic bands/tubing, hand weights, and body-weight exercises) versus usual care/equipment PR	6 to 28 weeks, session 20 to 60 minutes	6MWD 85 m (95% CI 37 to 132; I² = 86%), ISWT 168 m (95% CI 19 to 317), HRQoL -SMD 0.99 (95% CI 0.31 to 1.67), dyspnea (mMRC −1.3; 95% CI −1.8 to −0.8)	Minimal equipment PR produces measurable gains in exercise capacity and HRQoL versus usual care; no difference versus equipment PR.
De Lima et al., 2022, (33) Brazil	Review of 8 RCTs (n=332)	Stable adults	COPD	Elastic-resistance versus no/machine	8–12 weeks (3×/week)	Strength - knee extensors (SMD = 0.52; 95% CI = 0.09–0.95; I² = 40%), 6MWT (MD = 10.94 m; 95% CI = −8.65 to 30.53)	Elastic bands produce effects comparable to machines; effective low-cost option.
Fagian et al., 2024, (24) Uruguay	17 patients	Adults	COPD	Tai chi group versus control group (aerobic training, breathing exercises, resistance exercises, and relaxation), Tai chi group additional 20-minute Tai Chi	12 weeks (24 sessions)	PROMIS (p > 0.05), SGRQ1 month (44 ± 5 versus 46 ± 5; p = 0.742) and 3 months (44 ± 5 versus 40 ± 6; p = 0.916), BODE at 3 month Tai Chi (2 ± 0.6 versus 3 ± 0.4; p = 0.889)	Tai chi improved functional capacity, symptoms, and health-related quality of life after PR.
Frei A. et al., 2022, (31) Switzerland	HOMEX – 1 and HOMEX - 2 with 120 participants each (protocol)	Adults, GOLD II–IV	COPD	HOMEX minimal-equipment (elastic bands and a chair) + supervision + telephone coaching	12 months, 6 days/week 15–20 minutes/day	1° Outcome: CRQ-Dyspnea MIC = 0.5 points, and sample size SD of 0.9, with 80% power and α = 0.05. 2° Outcome: 6MWT, 1-min Sit-to-Stand, CRQ, CAT, HADS, exacerbations, cost-effectiveness	A long-term protocol is hypothesized to maintain or improve dyspnea, functional capacity, HRQoL, and physical activity.
Habib GMM et al., 2020, (13) UK	Review of 13 trials (n=661)	Adults in LMICs	COPD, post-TB	Low-resource PR with limited equipment	12 weeks home-based program 5 days/week	Improvements in dyspnea (mMRC from 3.1 ± 0.7 to 2.2 ± 0.6; p < 0.05) , functional capacity (6MWT from 312 ± 68 m to 356 ± 70 m; +44 m; p < 0.05) and quality of life (SGRQ from 56 ± 11 to 44 ± 10; −12 points; p < 0.05)	Low-cost PR program yielded measurable changes in symptoms, functional performance, and HRQoL using a structure feasible for low-resource settings
Holland et al., 2017, (27) Australia	166 participants	Stable adults	COPD	Home-based versus center PR	8 weeks (+12-mo)	Improvements in 6MWD (home: +29.4 m, 95% CI 13.8–45.0; center: +10.8 m, −4.5 to 26.2) between-group difference of +18.6 m (−3.3 to 40.7), CRQ-(home: +4.24; center: +2.68)	Home non-inferior center-program; neither model sustained benefits at 12 months
Holland et al., 2021, (17) United States, Australia, Canada, and Europe,	—	—	CRD	ATS/ERS “modern PR” workshop	—	Identification of 13 essential components of PR	Endorses home/tele-PR when trial-tested and quality assured
Hurst et al., 2021, (41) Australia	—	—	COPD/asthma	ATS LMIC implementation workshop	—	Policy and system-level recommendations	PR scale-up prioritized for LMICs
McKeoug Z et al., 2018, (22) Australia	14 participants	Stable adults	COPD	Sun-style tai chi (short-form)	8 weeks 2x/week	Improvement in 6MWT distance (95% CI) 28 m (-7 to 63), and SGRQ total score was -4 points (-9 to 0.4).	No physical activity level increase at 8 weeks versus control
Moy LM et al., 2021, (26) USA	91 adults	Adults GOLD 3–4	COPD	Tai chi versus usual care versus walking	24 weeks Tai chi (12 weeks -2x/week + 12 weeks – 1x/week and home exc. 3x/week	6MWD effect size 0.25 (95% CI −0.24–0.73; p = 0.10); proportion with 6MWD improvement: 64% versus 39% (p = 0.05); CRQ-Fatigue improvement 59% versus 31% (p = 0.02); CRQ-Mastery improvement 47% versus 20% (p = 0.01)	Tai Chi after PR is feasible and safe, and maintenance of PR gains
Ochman M et al., 2018, (28) Poland	40 (22 Nordic walking)	Transplant candidates	ILD/COPD	Nordic walking PR (hospital+home)	12 weeks 6-week cycles (2 weeks hospital + 4 weeks home training)	Improvement in the distance 6-MWT (310 m – to 374 m, P = .0378), dyspnea MRC (P = .002), quality of life PCS (p = 0.039), and pain perception (P = .034) in the intervention group.	Nordic Walking improves functional capacity, dyspnea perception, and quality of life, with no adverse events reported.
Patel S et al., 2021, (32) UK	318+318 matched participants	Adults - GOLD A–D	COPD	Minimal-equipment versus gym-based PR	16 supervised sessions 8 weeks (2×/week)	ISW −3.1 m (−15.6 to 9.4), p = 0.63, CRQ-dyspnea +1.6 (0.4–2.8), *p = 0.009*, completion PR-gym (73.0%, *p = 0.014)*	Minimal equipment PR non-inferior ISW/CRQ; completion lower with minimal equipment.
Polkey MI, 2018, (35) China/UK	120 participants	Bronchodilator--naïve	COPD	Tai chi: 24-form Yang style, 1 h/day, 5 days/week, 12 weeks versus center PR: aerobic + resistance training, 1 h/session, 3 days/week, 12 weeks	2-week run-in, 12-week intervention, 12-week follow-up	SGRQ 4.5 points (95% CI 1.9–7.0; p < 0.001), 6MWD **–**22.7 m (95% CI –34.6 to –10.9; p < 0.001), mMRC 0.32 (95% CI 0.15–0.49; p < 0.001)	Tai Chi is a viable, low-equipment modality whose benefits persist longer after supervised training compared to PR.
Pothirat C, 2015, (34) Thailand	30 patients	Advanced COPD (GOLD II–IV)	COPD	Community/home PR (walking, breathing, strength)	8 weeks supervised 2x/week, strength training (dumbbells, sandbags, and theraband) and walking-based endurance training + 10-12 month home-based	6MWD 307.9 ± 87.4 m to 394.4 ± 143.6 m (P < 0.001; MCID reached from month 2 onward), SGRQ (52.1±13.0 to 31.3±20.6), mMRC (3.1 ± 0.9 to 2.3±1.2)	Low-cost PR sustained improvements across 12 months in muscle strength, 6MWD, SGRQ, and dyspnea
Pradella CO, 2015, (25) Brazil	44 participants	Adults - GOLD (mild to severe)	COPD	Home PR (walking, stairs, upper limb exercises -1-kg oil can) + calls	24-session home-based 8 weeks 3x/week	The home PR group presented improvement in 6MWT 65.7 ± 83.1 m (p = .039) compared with 5.5 ± 92.9 m in control group, endurance 316.6 ± 81.8 m (p < .001), SGRQ	Home-based PR using minimal equipment is just as effective as traditional PR and is a viable alternative in low-cost contexts
Ramos EMC, 2014, (36) Brazil	45 participants	Adults - Moderate COPD	COPD	Elastic tubing(ET): Upper and lower limb resistance Lemgruber® elastic tubes versus Conventional training(CT): weights machines (supervised)	24 sessions 8 weeks, 3x/week, 60 minutes	6MWT CT: +42.2 m (95%CI 11.7–72.7); ETT: +73.4 m (95%CI 37.4–109.4) (p < 0.05.0), strength and quality of life improved in all domains in both groups (p < 0.05),	ET training elicited greater 6MWD improvement than conventional training; both increased strength/CRDQ.
Rochester CL, 2023, (37) USA Canada Australia UK	82 RCTs	Adults	COPD/ILD/PH	Center and tele/home PR	4 weeks –2 months	COPD 6MWD (mean difference 43.93 m, 95% CI 32.64–55.21), ISWD +39.77 m (22.38–57.15), dyspnea (+0.72 points, 0.19–1.25), HRQoL (−6.8 points, −9.26 to −4.52), SGRQ −6.8 points (−9.26 to −4.52)	Strong recommendations PR in stable COPD, post-exacerbation COPD, and ILD; conditional recommendation in PH; strong recommendation for both center-based PR and telerehabilitation as equivalent options.

Abbreviations: RCTs (Randomized Clinical Trial); UK (United Kingdom); USA (United States of America); PR (pulmonary rehabilitation); COPD (chronic obstructive pulmonary disease); CRD (chronic respiratory disease); HRQoL (health-related quality of life); 6MWD/6MWT (six-minute walk distance/test); ISWT (incremental shuttle walk test); SGRQ (St George’s Respiratory Questionnaire); CAT (COPD Assessment Test); CCQ (Clinical COPD Questionnaire); PHQ-9 (Patient Health Questionnaire-9); BODE (Body mass index–Obstruction–Dyspnea–Exercise).

## Results

### Evidence on effectiveness

This narrative review identified 20 publications evaluating low-cost PR across chronic respiratory diseases, although most studies focused on COPD (12, 30–32). The included evidence comprised randomized controlled trials, quasi-experimental studies, systematic reviews, clinical guidelines, and implementation reports conducted in high-, middle-, and low-income settings (12, 20, 22, 24, 30–35). Low-cost PR programs were delivered in home-based, community-based, primary healthcare, hospital outpatient, and specialized rehabilitation settings, reflecting substantial variation in service organization and resource availability (13, 20, 24, 33, 34).

Although COPD remains the dominant condition represented in low-cost PR research, this focus limits the applicability of current evidence to the broader burden of chronic respiratory disability in resource-constrained settings (22, 23, 36). In LMICs, rehabilitation needs may extend to individuals with post-tuberculosis respiratory impairment, bronchiectasis, post-COVID respiratory sequelae, diffuse parenchymal or interstitial lung diseases, pulmonary hypertension, asthma, occupational lung diseases, and mixed respiratory conditions. These conditions should be considered priorities for future investigation rather than interpreted as being adequately represented among the 20 included publications (23, 24, 36). This limitation is clinically relevant because real-world PR services may manage heterogeneous CRD populations; Rodrigues et al., for example, reported that only 39% of participants in a Brazilian PR cohort had COPD (36).

The methodological profile of the included literature was heterogeneous. Randomized controlled trials contributed evidence of clinical effectiveness, whereas quasi-experimental and implementation studies provided information on feasibility, adherence, and real-world delivery (12, 20, 21, 24, 30–35). Randomized and controlled studies generally reported improvements in functional exercise capacity, symptoms, functionality, and health-related quality of life following low-cost PR interventions (12, 20, 30–35, 37–39). Review-level evidence suggested that minimal-equipment or low-cost PR can provide clinically relevant effects when aerobic training, resistance training, education, and monitoring are adequately structured (13, 22, 23, 40). Several methodological limitations were identified, including moderate methodological quality in some trials, small samples, single-center designs, short intervention periods, and unavoidable performance bias associated with the difficulty of blinding participants and therapists in exercise interventions (12, 13, 22, 30, 33–35, 40).

The intervention components most frequently reported in low-cost PR included walking, elastic resistance, improvised weights, bodyweight exercise, chairs, step-based activities, and structured education for self-management (18, 20, 21, 31, 32, 34, 35, 37–39). Holland et al. described essential components of modern PR, including exercise training, education, self-management support, and strategies aimed at long-term behavior change (18). Walking was frequently used as an aerobic modality because it is inexpensive, accessible, and adaptable to home or community settings (12, 21, 31, 35). Although many studies included individual PR components, fewer programs combined aerobic and resistance exercise according to guideline-based recommendations, with relevant examples including Anastasaki et al. (33), Patel et al. (41), and Pradella et al. (34). Session frequency and duration ranged from two to six sessions per week and from 20 to 60 min per session, reflecting adaptations to patient needs, supervision models, and local resource constraints (13, 20, 22).

Across the included studies, functional capacity improved when evaluated using the six-minute walk test (6MWT) and incremental shuttle walk test (ISWT), while health-related quality of life improved when assessed using the St. George's Respiratory Questionnaire (SGRQ), COPD Assessment Test (CAT), and Chronic Respiratory Questionnaire (CRQ) (12, 20–22, 34, 35, 37–42). Several studies reported changes that met or exceeded commonly used minimal clinically important difference thresholds, including approximately +30 m for the 6MWT, −4 points for the SGRQ, −2 points for the CAT, and +0.5 points for CRQ domains (13, 20–22, 33, 40).

In community- and home-based programs using minimal equipment, Ramos et al. (39) reported a 6MWT gain of +73.4 m with elastic tubing training. Pothirat et al. (31) reported a between-group difference of +65.7 m and, in a long-term community model, an increase from 307.9 ± 87.4 to 394.4 ± 143.6 m, with sustained benefits at 12 months. Ochman et al. (38) reported an increase in 6MWT distance from 310 to 374 m following a low-cost rehabilitation strategy.

Tai Chi-based and home-based programs also produced clinically relevant functional and health-related quality-of-life benefits, suggesting that culturally adaptable and low-resource exercise modalities may complement conventional center-based PR (20, 30, 32, 34, 43). Polkey et al. (32) reported favorable outcomes following Tai Chi, while home-based studies demonstrated non-inferior functional or quality-of-life outcomes compared with center-based PR when appropriate instruction and follow-up were provided (20, 34).

Improvements in health-related quality of life were observed across low-cost PR studies, with reported SGRQ reductions of approximately −6.4 to −9.3 points and CAT reductions of approximately −4 to −6 points (13, 21, 33). CRQ domains improved by approximately 0.6–1.0 points, reaching the commonly used 0.5-point threshold in relevant studies (20). When minimal-equipment PR was compared with conventional or specialist-equipment PR, differences generally remained within non-inferiority margins for functional outcomes, while simple-equipment resistance training produced clinically relevant strength gains (22, 40, 41).

Overall, the available evidence indicates that low-cost PR can improve functional capacity, symptoms, and health-related quality of life, particularly among people with COPD, when programs include structured exercise prescription, education, progression, and monitoring (12, 20, 22, 23, 31–35, 39–41). Nevertheless, the evidence base remains limited by COPD predominance, heterogeneity of intervention protocols, variable supervision models, relatively little primary care research, scarce cost-effectiveness evaluation, and insufficient long-term sustainability data (18, 22–24, 36, 40).

### Implementation models

Implementation of low-cost PR has been reported in home-based, community-based, primary care, and specialized outpatient contexts, reflecting adaptation to local resources and health-system capacities (20, 25, 33, 35). Home-based interventions frequently involved walking, elastic resistance, bodyweight exercises, or improvised equipment and were delivered with less center-based professional contact than conventional PR (20, 35).

Nevertheless, the feasibility of home-based PR should not be assumed across all low-resource settings, because adherence and safety may be affected by household, environmental, cultural, and socioeconomic constraints (16, 25). Unsafe walking environments, overcrowded housing, limited privacy, low health literacy, gender-related restrictions, air pollution, climate extremes, family responsibilities, and competing socioeconomic priorities may limit participation in home-based PR (25). Therefore, home-based PR should include context-specific feasibility assessment, flexible exercise options, caregiver involvement when appropriate, safety screening, symptom monitoring, and locally acceptable strategies for exercise progression (20, 23, 25).

Low-cost programs delivered outside specialized centers may reduce geographical and financial barriers and may require less frequent face-to-face professional contact (12, 13, 20, 31, 34, 35). Reduced professional contact, however, should not be interpreted as absence of professional oversight, because PR requires appropriate assessment, individualized prescription, exercise progression, monitoring, and referral pathways for clinical deterioration (18, 23–25).

Sustainable low-cost PR may involve structured task-sharing with physiotherapy assistants, community health workers, primary care nurses, peer trainers, and family caregivers, where such roles are legally recognized and appropriately supervised (23–26, 44). Depending on local organization and professional regulations, community health workers, primary care nurses, rehabilitation assistants, peer supporters, or family caregivers may contribute to education, adherence support, follow-up, and reinforcement of prescribed activities under appropriate supervision (23–26). In Brazil, however, physiotherapeutic methods and techniques are legally reserved for licensed physiotherapists; therefore, PR assessment, prescription, progression, and clinical decision-making should remain physiotherapist-led (44, 45).

## Discussion

### Research gaps and perspectives

This narrative review indicates that low-cost PR has the potential to improve exercise capacity, symptoms, and quality of life among individuals with COPD and other CRD (21, 22, 30, 42). The predominance of relatively recent publications also reflects growing interest in approaches that reduce dependence on specialized equipment and infrastructure (22–24, 30, 35, 40).

Overall, low-cost PR can achieve outcomes comparable to conventional programs when exercise prescription, education, safety, progression, and monitoring are adequately structured (12, 20, 22, 23). Supervision appears to contribute to adherence and exercise quality, while limited access to rehabilitation remains an important barrier to participation and outcomes (24, 25, 33, 35).

A major limitation of the available evidence is the predominance of studies involving people with COPD, despite the broader spectrum of CRD associated with exercise limitation, dyspnea, reduced participation, and impaired quality of life (22, 23, 36). This predominance restricts generalizability to the broader spectrum of respiratory disability encountered in clinical practice, particularly in LMICs (23, 24, 36). Future low-cost PR research should therefore explicitly examine populations with other high-burden CRD, including post-tuberculosis respiratory impairment, bronchiectasis, post-COVID respiratory sequelae, interstitial or diffuse parenchymal lung disease, and pulmonary hypertension. These conditions are proposed here as priorities for future research in response to the limited disease representation identified in the reviewed evidence, rather than as conditions adequately evaluated among the 20 included publications (23, 24, 36).

Another important consideration is that evidence supporting home-based PR should not be generalized without considering the environment in which patients are expected to exercise (16, 20, 25). In resource-constrained settings, potential barriers may include unsafe walking spaces, limited household space or privacy, low health literacy, family or caregiving responsibilities, environmental exposures, socioeconomic constraints, and other locally relevant cultural or social factors (16, 25). Future implementation studies should therefore assess these contextual determinants explicitly when evaluating feasibility and adherence.

Implementation in LMICs requires adaptation to workforce availability and local health-system capacity, because conventional specialist-led PR models may be difficult to scale in settings with limited rehabilitation infrastructure (23–26).

Decentralized supervision, task-sharing, community health worker involvement, peer support, family-supported rehabilitation, and periodic professional review may provide feasible alternatives when specialist rehabilitation teams are scarce (23–26).

Remote and hybrid monitoring strategies, including telemonitoring and telerehabilitation, may contribute to expanded access, particularly in rural or geographically remote settings (17, 18, 23, 46). However, digital interventions should be adapted to actual patterns of access and literacy because unstable internet connectivity, shared devices, and limited digital skills may restrict participation (25, 46). Where appropriate, lower-bandwidth strategies such as telephone follow-up, text-based communication, asynchronous instructions, printed materials, and caregiver-supported participation may provide practical alternatives to exclusively high-bandwidth platforms (25, 46).

Primary healthcare represents another important implementation setting. Low-cost PR can be delivered within primary healthcare when guided by structured assessment, individualized exercise prescription, education, safety procedures, and follow-up strategies (23, 24, 33, 35). Meaningful integration, however, also requires clear referral and communication pathways between primary care, chronic respiratory disease services, noncommunicable disease programs, hospital discharge pathways, community services, and, where locally relevant, TB/post-TB care (24, 27, 33). Simplified referral processes, feedback to referring clinicians, and continuity after completion of rehabilitation may improve integration and uptake (24, 27, 33).

Behavioral and educational components should remain central to low-cost PR because the benefits of exercise are unlikely to be sustained without self-management, adherence support, and long-term behavior change (18, 23, 25, 47). Programs should provide culturally and linguistically appropriate education addressing symptom monitoring, recognition of clinical deterioration, pacing, physical activity, medication use when relevant, health beliefs, stigma, family engagement, and patient expectations (18, 23, 25, 26, 47).

Equity should also be considered explicitly. Reducing the cost of exercise equipment does not necessarily remove transportation costs, travel requirements, loss of work time, caregiving responsibilities, disability-related barriers, out-of-pocket expenses, or geographical inequalities (24, 25, 27, 36). Future implementation research should assess whether low-cost models actually reach rural, socioeconomically disadvantaged, disabled, vulnerable, or marginalized populations and whether patient and household costs remain barriers despite lower program-level resource requirements.

Long-term sustainability remains insufficiently evaluated. The included studies provide limited evidence regarding cost-effectiveness, resource allocation, and sustainability beyond the intervention period (22, 24, 40). Sustainable implementation may depend on workforce preparation, integration into routine chronic care, stable referral pathways, local adaptation, maintenance rehabilitation, community ownership, and financing models that do not rely exclusively on temporary external resources (24–27). Public, community, academic, or public-private arrangements may warrant evaluation where locally appropriate, but their contribution to sustainability should be assessed rather than assumed.

Future studies should therefore evaluate not only clinical outcomes but also implementation outcomes such as reach, adoption, feasibility, fidelity, acceptability, cost, equity, continuity, and sustainability. However, collectively, the evidence supports a broader interpretation of low-cost PR as a context-adapted rehabilitation strategy, rather than simply an exercise program that uses inexpensive equipment (18, 23–25). Minimal equipment may reduce one barrier to access, but sustainable expansion also requires appropriate workforce models, referral pathways, primary healthcare integration, culturally responsive education, context-sensitive remote delivery, equitable access, and continuity of care (18, 23–27, 46).

## Conclusion

This narrative review indicates that low-cost PR can provide meaningful improvements in functional capacity, symptoms, and health-related quality of life, with several home-, community-, and minimal-equipment approaches demonstrating outcomes comparable to conventional PR. However, the evidence remains heterogeneous, with methodological limitations, a predominance of COPD-focused studies, limited representation of other CRD, and scarce evidence regarding cost-effectiveness and long-term sustainability.

Low-cost PR should therefore not be interpreted solely as rehabilitation performed with inexpensive equipment. Rather, it may be understood as a context-adapted approach that preserves essential PR components while adapting delivery to local infrastructure, workforce capacity, healthcare organization, and patient circumstances.

Future studies should examine broader CRD populations and evaluate implementation outcomes in addition to clinical effectiveness. Integration within primary healthcare, structured education, locally appropriate task-sharing, context-adapted referral and follow-up pathways, maintenance strategies, and explicit consideration of equity may represent important components of future low-cost PR models.
